# Genetic and phenotypic diversity of selected Kenyan mung bean (*Vigna radiata* L. Wilckzek) genotypes

**DOI:** 10.1186/s43141-021-00245-9

**Published:** 2021-09-27

**Authors:** Jedidah Wangari Mwangi, Oduor Richard Okoth, Muchemi Peterson Kariuki, Ngugi Mathew Piero

**Affiliations:** 1 Department of Biochemistry, Microbiology and Biotechnology Kenyatta University, P.O. Box 43844-00100, Nairobi, Kenya; 2 Department of Plant Science, Kenyatta University, P.O. Box 43844-00100, Nairobi, Kenya

**Keywords:** Mung beans, Gene diversity, Phenotypic characterization, SSR

## Abstract

**Background:**

Mung bean is a pulse crop principally grown in the tropic and subtropic parts of the world for its nutrient-rich seeds. Seven mung beans accessions from Eastern Kenya were evaluated using thirteen phenotypic traits. In addition, 10 SSR markers were used to determine their genetic diversity and population structure. This aimed at enhancing germplasm utilization for subsequent mung bean breeding programs.

**Results:**

Analysis of variance for most of the phenology traits showed significant variation, with the yield traits recording the highest. The first three principal components (PC) explained 83.4% of the overall phenotypic variation, with the highest (PC1) being due to variation of majority of the traits studied such as pod length, plant height, and seeds per pod. The dendogram revealed that the improved genotypes had common ancestry with the local landraces. The seven mung beans were also genotyped using 10 microsatellite markers, eight of which showed clear and consistent amplification profiles with scorable polymorphisms in all the studied genotypes. Genetic diversity, allele number, and polymorphic information content (PIC) were determined using powermarker (version 3.25) and phylogenetic tree constructed using DARWIN version 6.0.12. Analysis of molecular variance (AMOVA) was calculated using GenALEx version 6.5. A total of 23 alleles were detected from the seven genotypes on all the chromosomes studied with an average of 2.875 across the loci. The PIC values ranged from 0.1224 (CEDG056) to 0.5918 (CEDG092) with a mean of 0.3724. Among the markers, CEDG092 was highly informative while the rest were reasonably informative except CEDG056, which was less informative. Gene diversity ranged from 0.1836 (CEDG050) to 0.5102 (CDED088) with an average of 0.3534. The Jaccards dissimilarity matrix indicated that genotypes VC614850 and N26 had the highest level of dissimilarity while VC637245 and N26 had lowest dissimilarity index. The phylogenetic tree grouped the genotypes into three clusters as revealed by population structure analysis (*K* = 3), with cluster III having one unique genotype (VC6137B) only. AMOVA indicated that the highest variation (99%) was between individual genotype. In addition, marker traits association analysis revealed 18 significant associations (*P* < 0.05).

**Conclusion:**

These findings indicate sufficient variation among the studied genotypes that can be considered for germplasm breeding programs.

**Supplementary Information:**

The online version contains supplementary material available at 10.1186/s43141-021-00245-9.

## Background

Mung bean is mainly produced in the tropical and the subtropical parts of the world and it adapts well to the cropping systems practiced in these regions [1]. It is highly preferred due to its rapid growth, early maturity, and its ability to fix nitrogen. In Kenya, it is mainly grown in eastern and western region where it yield per hectare is low, 42,321 t/ha as at 2019 [2]. Besides, there is scanty information on the genetic diversity of mung bean germplasm due to limited genomic research done as compared to other *Fabeaceae* family species. It belongs to the genus *Vigna* that has over 100 plant species [3]. Mung bean production has been based on local landraces until the twentieth century, though its improvement has been minimal [4]. Globally, food insecurity is an issue of concern due to changing climate. However, mung bean, a pulse crop has proven to be a protein-rich food crop (24%) hence an important food crop [5]. When sprouted, it is a rich source of iron, calcium, and vitamin C [6].

Conventional breeding has delayed the generation of new mung bean cultivars that bear quality yield contributing traits [7]. Therefore, it is necessary that accurate information on the genetic diversity of mung bean collections preserved in the germplasm be availed to ensure proper germplasm utilization and subsequently the success of mung bean crop breeding programs [8]. This germplasm enhancement ensures that useful genes and their combinations are identified and used in various development programs [9]. Genetic diversity of mung beans in Kenya has not been done and this hampers the utilization of its genetic rich germplasm. Mung bean improvement can therefore play a key role in agricultural development, the main contributor to economic growth in Kenya, especially in the arid and semi-arid areas.

The invention of polymerase chain reaction (PCR) by Mullis [10] has enabled molecular characterization of organisms and hence their genetic improvement [11]. Development of many DNA markers has enabled germplasm characterization of the duplicated accessions, population structures, and genetic relationships and establishes distribution of variation between individuals and accessions. Marker choices that have been used in genetic diversity studies include RAPD [12], eSSR [13], SRAP [14], ISSR [15], STMS [16], and AFLP [17]. Microsatellites have proven to be a highly attractive genetic marker since they are highly reproducible, polymorphic, multi-allelic, co-dominant, and therefore considered for this study [18]. In this study, genetic characterization was done using ten SSR markers which have also been successfully used to study the diversity of other crops like *Vigna vexillata* [19].

Mung beans prefer well-drained loam soil, which is fertile and with a PH value range of 5.5-8.2 for maximum yield. However, abiotic and biotic stresses coupled with limited genetic diversity contribute to a low harvest index. Characterization of mung beans is of immense value and with the development of mung bean genetic linkage map, identification of the quantitative trait loci (QTLs) can be done with ease [20]. Genetic characterization ensures selection of a divergent parent for hybridization. Polymorphic markers enable genetic mapping of the yield contributing QTLs that can subsequently be used in genetic breeding programs. The objectives of this study were (1) to establish the genetic diversity of mung beans from Eastern Kenya using simple sequence repeat markers and (2) to determine the phenotypic variation of the mung bean genotypes and the correlation between yield traits.

## Methods

### Plant material collection and preparation

Seven mung bean genotypes grown in Machakos, Embu, and Tharaka Nithi counties in Eastern Kenya were obtained from Embu, Kenya Agricultural Livestock and Research Organization (KALRO). They are KPS1, N26, VC637245, VC61753B, VC6173B, VC614850, and VC6137B. These genotypes were selected based on farmer’s preference, drought resistance, and high yield. The seeds were then taken to Kenyatta University Plant Transformation Laboratory. Six seeds per genotype were planted in a pot with three replications per genotype following a randomized complete block design (RCBD). They were watered daily and germinated between 5 and 7 days.

### Phenotypic characterization

Morphological characterization of three plants per pot per genotype, selected randomly, was done at 6 weeks, 8 weeks, and at maturity using quantitative morpho-agronomic descriptors developed by Bioversity International [21]. The quantitative traits studied included stem height, leaf length, leaf width, leaf diameter, leaf petiole diameter, pods per plant, days to maturity, pod length, seed yield, harvest index, and biological yield per plant. A hundred seeds per plant were weighed using a weighing balance to determine the yield variation among the genotypes. Measurements were taken in triplicates and the means standardized before analysis. Biological yield was determined by weighing the whole plant in the laboratory using a weighing balance. The harvest index was calculated by dividing the seed weight per plant by its biological yield.

### Molecular characterization

Leaf samples from 21-day old mung beans were harvested, 100 mg per plant, frozen in liquid nitrogen and stored at −80 °C for DNA extraction. Total genomic DNA was extracted using CTAB method with slight improvements [22], and the quality determined by 1% agarose gel electrophoresis. It was then diluted to 20 ng/μl and stored at −20 °C until use. Ten sets of simple sequence repeat (SSR) marker primers developed by Gunjeet [23] were used to amplify the extracted DNA. The primers were selected based on their annealing temperature and the amplicon size of at most 300 base pairs (Table 1). PCR was done in a total volume of 25 μl containing 2 μg (1μg/μl) genomic DNA and 0.5 μM of each primer. The master mix comprised 25 units/ml of Taq DNA polymerase, 22 mM KCl, 20 mM Tris-HCl (pH 8.9 at 25 °C), 1.8 mM MgCl_2_, 22 mM NH_4_Cl, 0.05% Tween® 20; 5%glycerol, 0.06% IGEPAL® CA 360, 0.2 mM of each dNTPs; of each primer of DNA template and of One Taq DNA Polymerase. The PCR amplification programs were done in a thermo cycler with a cycle profile comprising initial denaturation for 4 min at 94 °C; 35 cycles of denaturation at 94 °C for 15 s, annealing at an average annealing temperature for the primers ranging from 44-55 °C for 60 s, extension at 68 °C for 45 s, and final hold at 72 °C for 10 min. The obtained PCR products were analyzed by electrophoresis on 2% (2g of agarose in 100 ml of 1X TAE), agarose gel with a 100 base pair ladder and then viewed under UV Trans-illuminator and the gel photos taken using a digital camera. One hundred base pair DNA ladder was used to determine the molecular weight of the PCR products.

**Table 1:** SSR primers used and their sequences

Primer name	Forward primer sequence (5′ to 3′)	Reverse primer sequence (3′ to 5′)	Repeat motif	Estimated base pair
CEDG006	AATTGCTCTCGAACCAGCTC	GGTGTACAAGTGTGTGCAAG	(AG)10AA(AG)18	140-162
CEDG010	TGGGCTACCAACTTTTCCTC	TGAGCGACATCTTCAACACG	(AG)21	180-210
CEDG050	GGCAGAATCGTACAAGTG	GTCAGATTCTCGCTTGCATG	(AG)12	140-160
CEDG056	GAACTTAACTTGGGTTGTCTGC	GCTATGATGGAAGAGGGCATGG	(AG)14	150-264
CEDG088	TCTTGTCATTTAGCACTTAGCACG	CTACCTATCTGAGGGACAC	(AG)7	110-140
CEDG092	TCTTTTGGTTGTAGCAGGATGAAC	TACAACTGATATGCAACGGTTAGG	(AG)17	140-220
CEDG214	CACTCACTGCAAAGAGCAAC	CTACCTATCTGAGGGACAC	(AG)4 AA (AG)31	180-205
CEDG232	GATGACCAAGGTAACGTG	GGACAGATCCAAAACGTG	(AG)16	205
CEDG253z	CACTTCCATGATGACTCACC	CACCCTTCTTTATCCTCTTCG	(AG)30	253
CEDG305	GCAGCTTCACATGCATAGTAC	GAACTTAACTTGGGTTGTCTGC	(AG)22	106-130

### Data analysis

Data was analyzed descriptively and through one-way ANOVA using the Minitab software version 19.2. Tukey’s post hoc was done to compare means at a 95% confidence level. Cluster analysis yielded a dendrogram constructed from the mean values of all the phenotypic traits across the genotypes. Principal component analysis was done to evaluate each character’s contribution to the overall observed phenotypic diversity using the Minitab software (version 19.2).

The observed bands were all scored based on the binary coding system: presence (1) or absence (0) of the amplification fragments using SSR primers. The obtained matrix was subjected to power marker software to analyze each mung bean based on the allele number, polymorphic information content, gene diversity, and major allele frequencies. Power marker (version 3.25) was used to compute the polymorphism information content (PIC) and the extent of gene diversity. Unweight pair group method with the arithmetic average (UPGMA) clustering method was used to convert the dissimilarity matrix values into a dendrogram. Analysis of molecular variance (AMOVA) was done using GenALEx to determine the variations among and within populations from the regions. GenALEx software was used to generate a two-dimensional representation (PCoA) of the genetic relationship among the studied genotypes. Association analysis, TASSEL software version 5.0 was used for this analysis. Genetic linear model (GLM) was used to determine the association between the phenotypic and genetic analysis. The markers were defined as being significantly associated to the phenotypic traits based on the significant association threshold for GLM. Population structure analysis was done using structure software, where we performed values for nine runs. Genetic differentiation (fixation index Fst) was done among the three populations using R package.

## Results

### Phenotypic characterization

The studied phenotypic traits of green grams varied significantly across the seven genotypes except for the petiole width (*P* > 0.05; Table 2). The average number of pods per plant, seed leaf length, and leaf length recorded the highest variation among the thirteen studied traits. Seed leaf length showed moderate variation with genotype VC614850 having the highest length of 8.4 cm. There was no significant variation (*P* > 0.05) in petiole width among the genotypes studied which recorded a range of 0.028. However, petiole diameter had significant variation with genotype VC637245 having the highest length of 0.142 mm. Plant height recorded significant variation with genotype VC6173B being the tallest while all the genotypes recorded a range of 10.32. Among them, KPS1, N26, VC61753B, and VC6173B genotypes were the tallest. Additionally, significant variation was noted in the leaf length with VC6137B having the highest mean value of 8.40 cm.

**Table 2:** Morpho-agronomic traits for the 7 *Vigna radiata* genotypes

Genotypes traits	KPS1	N26	VC6137B	VC614850	VC6173B	VC61753B	VC637245
**Petiole width (cm)**	0.132 ± 0.004^a^	0.130 ± 0.005^a^	0.132 ± 0.004^a^	0.124 ± 0.004^a^	0.120 ± 0.004^a^	0.128 ± 0.004^a^	0.118 ± 0.004^a^
**Petiole diameter (cm)**	0.108 ± 0.003^b^	0.106 ± 0.004^b^	0.140 ± 0.003^a^	0.140 ± 0.003^a^	0.110 ± 0.004^b^	0.122 ± 0.007^ab^	0.142 ± 0.004^a^
**Seed leaf length (cm)**	8.14 ± 0.07^a^	8.10 ± 0.11^a^	7.50 ± 0.07^b^	8.44 ± 0.07^a^	8.24 ± 0.05^a^	8.28 ± 0.07^a^	8.12 ± 0.10^a^
**Plant height (cm)**	31.92 ± 0.65^a^	30.74 ± 0.45^ab^	22.66 ± 0.80^c^	27.72 ± 0.57^b^	32.98 ± 0.56^a^	31.14 ± 0.33^ab^	27.40 ± 0.64^b^
**Leaf length (cm)**	7.88 ± 0.21^abc^	6.78 ± 0.22^bc^	8.40 ± 0.14^a^	7.96 ± 0.26^ab^	6.54 ± 0.21^c^	7.16 ± 0.09^abc^	6.58 ± 0.23^c^
**Leaf diameter**	3.82 ± 0.04^b^	4.32 ± 0.07^a^	4.50 ± 0.04^a^	4.30 ± 0.05^a^	4.34 ± 0.02^a^	4.40 ± 0.03^a^	3.80 ± 0.05^b^
**Pod length (cm)**	6.14 ± 0.10^b^	7.12 ± 0.23^ab^	7.08 ± 0.25^ab^	6.86 ± 0.24^ab^	7.40 ± 0.20^ab^	7.96 ± 0.23^a^	7.52 ± 0.27^ab^
**Pods per plant**	20.00 ± 0.71^a^	16.60 ± 0.51^ab^	8.00 ± 0.63^d^	13.80 ± 0.74^bc^	13.20 ± 0.58^bc^	16.80 ± 0.80^ab^	11.60 ± 0.40^cd^
**Seeds per pod**	5.40 ± 0.25^b^	6.40 ± 0.60^ab^	4.00 ± 0.32^b^	6.20 ± 0.58^b^	7.00 ± 0.63^ab^	9.60 ± 0.51^a^	4.00 ± 0.32^b^
**Seed weight per plant (g)**	3.22 ± 0.28^b^	3.42 ± 0.34^ab^	2.75 ± 0.15^b^	3.07 ± 0.04^b^	3.11 ± 0.13^b^	4.94 ± 0.28^a^	2.02 ± 0.24^b^
**Plant dry weight (g)**	9.30 ± 0.70^a^	4.75 ± 0.57^b^	7.44 ± 0.32^ab^	9.49 ± 0.52^a^	5.27 ± 0.47^b^	7.51 ± 0.57^ab^	4.66 ± 0.37^b^
**Harvest index**	23.33 ± 0.55^d^	38.11 ± 0.72^a^	27.48 ± 0.64^c^	33.85 ± 0.46^b^	35.10 ± 0.07^ab^	35.57 ± 0.45^ab^	29.75 ± 0.95^c^
**Biological yield**	13.71 ± 0.94^a^	8.97 ± 0.86^b^	9.99 ± 0.43^ab^	9.07 ± 0.14^b^	8.87 ± 0.36^b^	13.85 ± 0.64^a^	6.74 ± 0.63^b^

Values expressed as mean ± standard error of the mean for three replicates. Means that do not share a superscript letter across the row are significantly different by one-way ANOVA following Tukey’s post hoc test (*p* < 0.05)

Moderate variation was seen in leaf diameter with VC6137B genotype having the widest leaf. Pod length showed the highest variation among the studied traits, with genotype VC61753B recording the longest pods while KPS1 had the lowest (Table 2). Significant variation was noted in the number of pods plant^−1^, with the improved genotype N26 having the highest number of pods. Number of seeds per pod differed significantly (*P* >0.05) among genotypes where the VC61753B genotype had the highest mean of 9.6 while genotypes VC6137B andVC637245 had the lowest mean of 4.0 (Table 2). Seed weight among the genotypes as well recorded significant variation (Table 2). Genotype VC637245, however, recorded the lowest mean value among all the genotypes. Highest plant dry weight was recorded in three genotypes VC61753B, KPS1, and VC614850. Significant variation was also noted in the harvest index with genotype N26 having the highest index (Table 2). Biological yield showed significant variation among the genotypes except VC61753B, KPS1, and VC614850 genotypes which were not significantly different.

### Cluster analysis

**Fig. 1:**
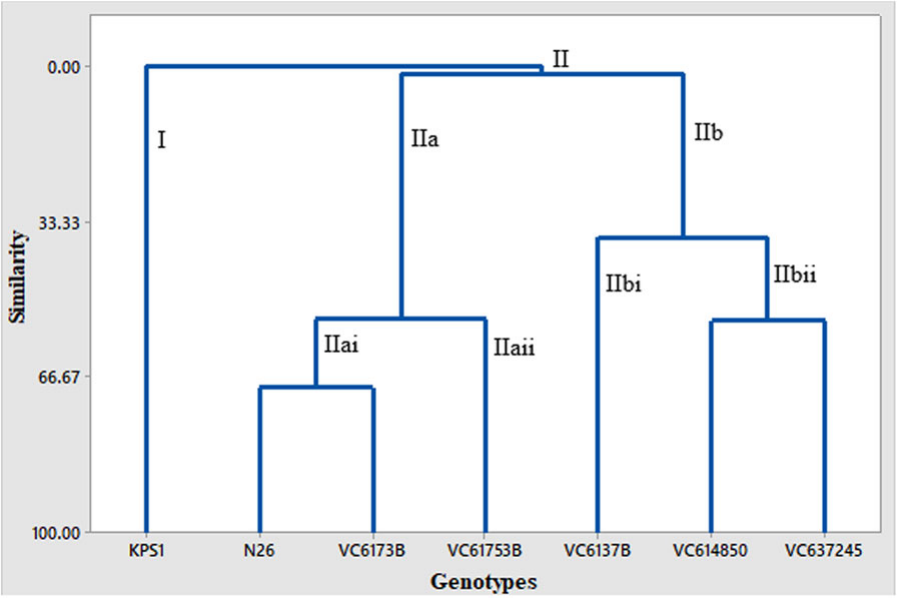
Neighbor joining dendogram showing morphological diversity of green gram genotypes

The genotypes were discriminated into two distinct phenotypic super clusters based on the 13 morpho-agronomic traits: cluster I and cluster II (Fig. 1). Cluster I comprised four varieties while cluster II had three mung bean varieties. Cluster I had two sub-clusters: Ia and Ib. Sub cluster Ia had KPS1 genotypes only whereas sub cluster Ib was more diverse and comprised three mung bean genotypes VC6137B, VC614850, and VC637245. It was further divided into sub cluster Ibi and Ibii where sub cluster Ibi had one genotype, VC6137B. Sub cluster Ibii comprised the other two varieties which are then segregated to form individual groups of genotypes VC614850 and VC637245. Sub cluster II comprised the other three genotypes both improved and landraces; N26, VC6173B, and VC61753B. The cluster was sub divided into two sub clusters, IIa and IIb, with the VC61753B variety forming its own group. The remaining two genotypes formed individual groups with a high similarity index.

### Principal component analysis

Principal component analysis (PCA) of the 13 morphological traits indicated phenotypic differentiations that play a key role in the phenotypic diversity of the studied green gram genotypes. The first and the second principal components accounted for 64.7% of the overall variation. Eleven of the phenotypic traits contributed significantly to the first principal component which includes petiole width, seed leaf length, plant height, pod length, seeds per pod, pods per plant, seed weight per plant, plant dry weight, biological yield, and harvest index. However, two traits, petiole diameter and leaf diameter, exhibited negative correlation. Six traits on the other hand which include petiole width, leaf diameter, seed weight per plant, pods per plant, plant dry weight, and biological yield contributed positively to PC2 and explained 29.1% of the total phenotypic variation. However, negative correlation was depicted by petiole diameter, seed leaf length, and the number of seeds per pod, plant height, pod length, and harvest index traits. Principal component 3 explained 18.6% of the overall observed variation with ten of the traits showing positive correlation: biological yield, harvest index, plant dry weight, seeds per pod, seed weight per plant, pod length, leaf length, leaf diameter, petiole width, and diameter. Plant height, seed leaf length, and pods per plant negatively correlated to PC3 (Table 3).

**Table 3:** Principal component analysis of 7 *Vigna radiata* genotypes based on 13 traits

	PC1	PC2	PC3
Eigen value	4.626	3.788	2.423
% Total variance	35.6	29.1	18.6
% Cumulative	35.6	64.7	83.4
**Traits**	**Eigen vectors**		
Petiole width (mm)	0.026	0.421	0.193
Petiole diameter	−0.336	−0.082	0.181
Seed leaf length	0.31	−0.111	−0.216
Plant height	0.407	−0.039	−0.273
Leaf length	−0.202	0.416	0.196
Leaf diameter	0.056	−0.028	0.585
Pod length	0.104	−0.359	0.339
Pods per plant	0.361	0.205	−0.281
Seeds per pod	0.421	−0.018	0.238
Seed weight per plant	0.377	0.138	0.312
Plant dry weight	0.001	0.427	0.047
Harvest index	0.248	−0.318	0.264
Biological yield	0.242	0.393	0.097

**Fig. 2:**
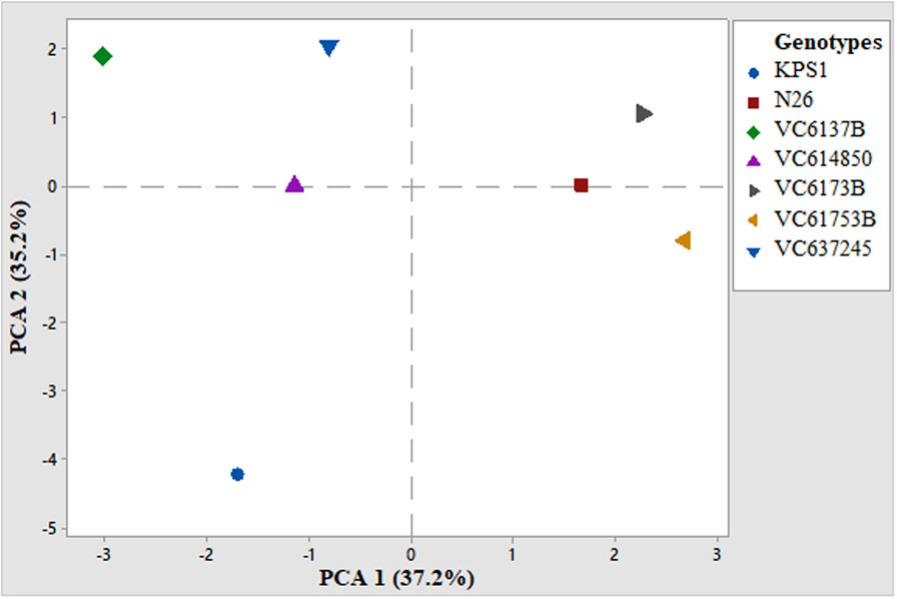
Principal component analysis (PCA) of mung bean genotypes based on phenotypic traits. The first two components had a variation of (1) 35.6% and (2) 29.1%

The scatter plot showed some variations among the studied mung beans which compared with clustering patterns shown in the neighbor joining dendogram for morphological traits. Principal components 1 and 2 accounted for much of the total variation (35.6%) and hence distinguished among the seven mung bean genotypes. The improved varieties fell in the second and the third quadrants and they clustered with the local landraces. Genotypes VC6137B and VC614850 clustered together in the first quadrant, while VC637245 clustered on its own in the fourth quadrant (Fig. 2).

### Gene diversity

Eight of the SSR markers depicted clear and consistent amplification profiles with all the mung bean genotypes. In contrast, two of the markers (CEDG232 and CEDG253) did not show any polymorphism with all the studied genotypes and gave band sizes of 200 bp and 150 bp, respectively. The capacity of the eight SSR markers to establish genetic diversity among the seven mung bean genotypes varied considerably. Analysis of the amplified products of mung bean accessions by the SSR markers indicated that the markers were marker indices per each polymorphic marker. The highest marker index was recorded in marker CEDG214 (0.73). The resolving power of each marker ranged from 0.18 in marker CEDG010 to 0.81 in marker CEDG214 with an average of 0.52 (Table 4).

**Table 4:** SSR markers used on the mung bean genotypes

Marker	BR	TB	PB	PPB	RP	MRP	MI
CEDG006	140-162	2	1	50	0.24	0.5175	0.18
CEDG010	180-210	2	1	50	0.18	0.5175	0.14
CEDG050	140-160	5	3	60	0.76	0.5175	0.32
CEDG056	150-164	3	3	100	0.62	0.5175	0.43
CEDG088	110-140	2	2	100	0.41	0.5175	0.21
CEDG092	140-220	2	2	100	0.38	0.5175	0.28
CEDG214	180-205	4	3	75	0.81	0.5175	0.73
CEDG305	106-130	3	3	100	0.74	0.5175	0.58
Average					0.52	0.5175	0.36

*BR* band range, *TB* total band, *PB* polymorphic band, *PPB* percentage polymorphic band, *RP* resolving power, *MRP* mean resolving power, *MI* marker index

A total of 23 alleles of the eight polymorphic SSR markers were recorded among genotypes which indicates the estimate of genetic diversity among the studied genotypes. The allele number for the individual markers ranged from 2 to 5 and the average was 2.875. The CEDG050 marker recorded the highest number of polymorphic alleles followed by CEDG214, CEDG056, and CEDG305 in that order. The highest allele frequency of 0.7152 was observed in CEDG056 marker while the least was recorded in CEDG050 and CEDG214 markers. The average major allele frequency was 0.5895 (Table 4). Null alleles were detected in some genotypes where no amplification product was detected in their combination. This conclusion was made after the experiment was repeated at least twice to ascertain the findings and rule out experimental error.

Twenty loci had null alleles in two to five of the studied mung bean genotypes. The genotypes that recorded the highest number of null alleles were VC614850, VC6173B, VC61753B, and N26. Four rare alleles were observed with the highest being recorded in CEDG050 followed by CEDG010 and CEDG092 markers. Gene diversity among the genotypes ranged from 0.5102 to 0.1836 with an average of 0.3534. The highest genetic diversity was recorded in CEDG010 and CEDG088 with the least being in CEDG214. Polymorphic information content calculated in each SSR locus reflected the level of polymorphism among the studied mung bean genotypes. It ranged from 0.1224 to 0.5918 with an average value of 0.3724. CEDG092 recorded the highest polymorphism while CEDG056 had the least polymorphism (Table 5). The PIC values were determined by allelic richness and the allele frequency among the studied genotypes whereby CEDG092 was found to be the most informative marker. The average polymorphism information content was 0.3724 which indicates that the markers are reasonably informative.

**Table 5:** The polymorphic SSR markers used with their allelic richness, allele frequency, gene diversity, and the PIC values

SSR markers	Linkage group	Allelic richness	Major allele frequency	Gene diversity	Homozygosity	PIC
CEDG006	2	2	0.5714	0.3265	0.0000	0.4898
CEDG010	3	2	0.7143	0.5102	0.0000	0.4082
CEDG050	2	5	0.4286	0.1836	0.0000	0.2857
CEDG056	9	3	0.7152	0.4600	0.0000	0.1224
CEDG088	4	2	0.7143	0.5102	0.0000	0.3061
CEDG092	8	2	0.5714	0.3265	0.0000	0.5918
CEDG214	1	4	0.4286	0.1836	0.0000	0.3673
CEDG305	3	3	0.5725	0.3265	0.0000	0.4081
Average		2.875	0.5895	0.3534	0.0000	0.3724

### Pairwise genetic dissimilarity analysis of seven mung bean varieties

This determined the genetic relatedness among the varieties by targeting the shared alleles. In this study, the coefficients ranged from 0.3464 to 0.6633 (Table 6). These dissimilarity coefficient values were used in the construction of the unweighted pair group method with arithmetic average as shown in Table 5. The genotypes VC637245 and N26 recorded the lowest dissimilarity value of 0.3464, whereas VC614850 and N26 had the highest level of dissimilarity (0.6633).

**Table 6:** Cord coefficients of dissimilarity among the pairs of seven green gram varieties

	KPS1	N26	VC637245	VC61753B	VC6173B	VC614850	VC6137B
KPS1	0.0000						
N26	0.5263	0.0000					
VC637245	0.5657	0.3464	0.0000				
VC61753B	0.5657	0.5263	0.4897	0.0000			
VC6173B	0.5657	0.5291	0.4899	0.4895	0.0000		
VC614850	0.3508	0.6633	0.5657	0.3509	0.5651	0.000	
VC61753B	0.4897	0.4472	0.4898	0.6325	0.6325	0.5657	0.0000

### Genetic differentiation of the mung bean populations

Genetic differentiation of the mung bean genotypes based on the counties they were obtained from indicated that there was variation of the genotypes based on the three different population. Highest variation was recorded between the genotypes from Machakos and Tharaka Nithi Counties (Table 7).

**Table 7:** Pairwise comparison of the three mung beans populations based on the Fst values

Embu	Tharaka	Machakos	
0.000	0.401	0.462	**Embu**
0.038	0.000	0.502	**Tharaka Nithi**
0.000	0.000	0.000	**Machakos**

### Clustering of the *Vigna radiata* genotypes using neighbor joining

**Fig. 3:**
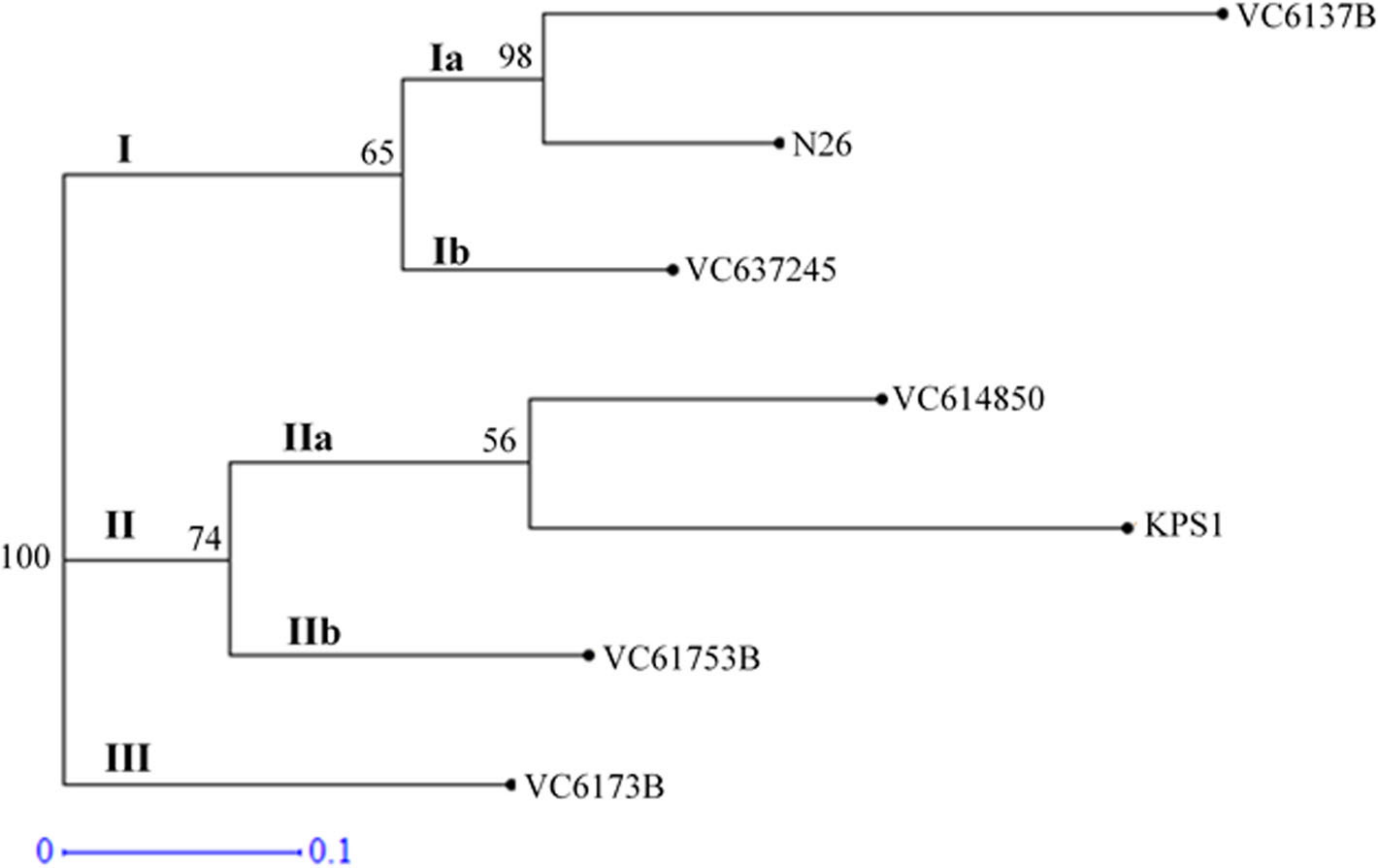
A phylogenetic tree showing the genetic relatedness between the 7 genotypes based on the ten microsatellite markers study

A phylogenetic tree revealed the genetic relatedness existing among the mung bean genotypes based on the eight microsatellite markers as shown by the bootstrap values. The seven genotypes were grouped into three main clusters (Fig. 3). Genotype VC6173B formed its own cluster labeled as cluster III hence distantly related with the rest of the genotypes (Fig. 3). Clusters I and II comprised 3 genotypes each, which were further subdivided into sub clusters. Genotype VC637245 formed its own sub cluster while genotypes N26 and VC6137B were grouped together. Cluster II comprised three genotypes that were further subdivided to form two sub clusters where genotype VC61753B formed its own sub cluster and the other two formed another sub cluster.

### Analysis of molecular variance (AMOVA)

Genetic variation among the genotypes was also determined using AMOVA. One percent of the variation partitioned among the genotype populations from the three regions with a *p* value of 0.622, whereas 99% (0.016) of the variation was within individuals (Table 8).

**Table 8:** Analysis of molecular variance (AMOVA); degrees of freedom (df), mean sum of squares (ss), mean of square deviation (MSD), percentage variation (%), and *p* values

Source	Df	SS	MSD	Est. var.	% variation	***p*** value
**Among pops**	2	3.833	4.833	0.028	1%	0.622
**Within pops**	7	11.333	2.833	2.833	99%	0.016
**Total**	9	19.167		2.358	100%	

### Principal coordinate analysis

**Fig. 4:**
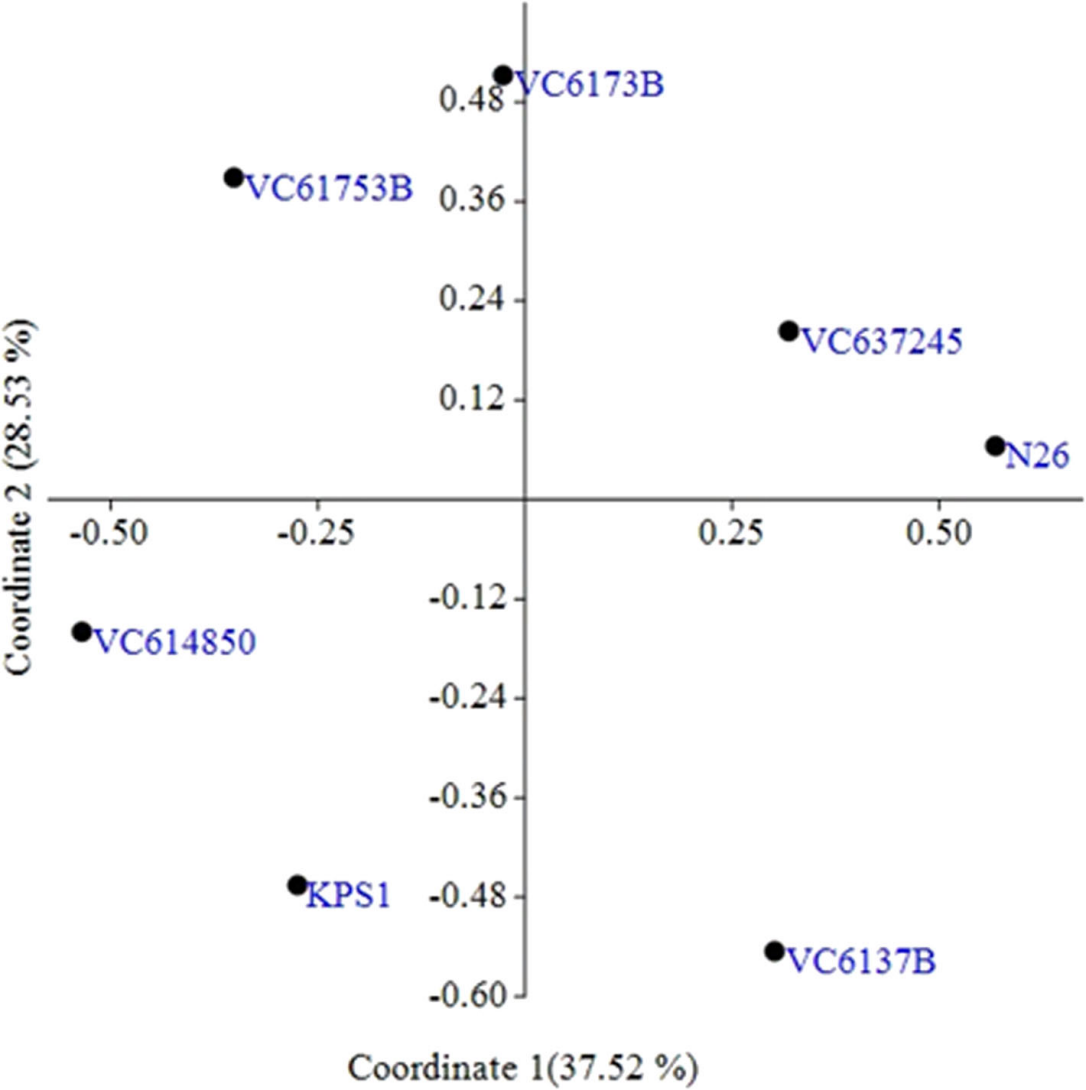
Principal coordinate analysis of *Vigna radiata* based on the ten simple sequence repeat markers. The percentage variations for the first two coordinates are 37.52% and 28.53%, respectively

PCoA analysis of the seven mung bean genotypes was used to show their genetic relatedness (Fig. 4). Principal coordinate 1 accounted for 37.52% relatedness whereas the second one accounted for 28.53%. All the seven genotypes were evenly distributed in the four quadrants. The first and the second principal coordinates cumulatively accounted for 66.05% of the overall genetic variation.

### Association analysis

Association analysis of the phenotypic traits revealed that majority of the alleles were associated to the traits. The association mapping identified 18 single-associated SSR markers associated with agronomic traits (*P* < 0.05). With regard to agronomic traits, five single-associated markers were associated with plant dry weight, four single-associated markers were associated with leaf length, two single-associated markers were associated with pod length, and seeds per pod respectively, and one single-associated marker was associated with seed leaf length, plant height, harvest index, and biological yields respectively (Table 9).

**Table 9:** Association of SSR markers with single agronomic trait of 7 *Vigna radiata* genotypes

Trait	Marker	marker_F	***p***	marker_Rsq	marker_df	marker_MS	Minor Obs
Sd leaf length	CEDG050b	26.70341	0.00356	0.84229	1	0.44434	1
Plant height	CEDG050b	9.74935	0.02618	0.661	1	50.24961	1
Leaf length	CEDG056a	37.25042	0.00171	0.88166	1	2.96439	3
Leaf length	CEDG010b	15.38572	0.01115	0.75473	1	2.53762	3
Leaf length	CEDG010c	15.38572	0.01115	0.75473	1	2.53762	3
Leaf length	CEDG050a	37.25042	0.00171	0.88166	1	2.96439	3
Leaf diameter	CEDG305c	8.27332	0.03474	0.6233	1	0.29762	3
Pod length	CEDG056a	6.56282	0.05054	0.56758	1	1.1155	3
Pod length	CEDG050a	6.56282	0.05054	0.56758	1	1.1155	3
Seeds per pod	CEDG010a	7.85387	0.03788	0.61101	1	13.72857	2
Seeds per pod	CEDG214d	7.90308	0.03749	0.6125	1	13.76191	3
Plant dry weight	CEDG056a	11.056	0.02089	0.68859	1	17.5086	3
Plant dry weight	CEDG056b	12.0241	0.0179	0.7063	1	17.95888	3
Plant dry weight	CEDG010b	27.40094	0.00337	0.84568	1	21.50298	3
Plant dry weight	CEDG010c	27.40094	0.00337	0.84568	1	21.50298	3
Plant dry weight	CEDG050a	11.056	0.02089	0.68859	1	17.5086	3
Harvest index	CEDG214d	21.57315	0.00561	0.81184	1	132.88	3
Biological yield	CEDG214a	31.70368	0.00245	0.86377	1	36.46101	2

*F F* value, *p p* value, *Rsq* regression square, *df* degree of freedom, *MS* mean square

The *p* values indicated a positive correlation between the genetic loci studied and the phenotypic markers used to characterize the mung bean genotypes.

### Population structure

**Fig. 5:**
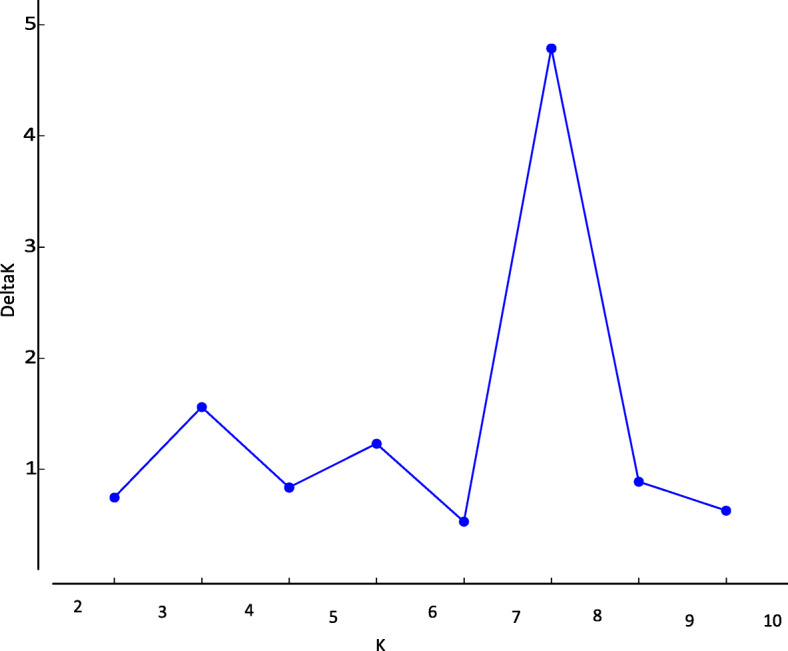
Population structure of seven mung bean genotypes based on SSR markers

Analysis of the mung bean genotypes’ genetic structure was done using the structure software. Ten runs were done by setting the number of populations to ten. The replication number was set to 100,000 and *K* = 2 converged well with higher likelihoods among runs. This aimed at identifying the accessions that represent the three populations. The delta *K* values ranged from 0.6 to 4.7 as shown in the graph (Fig. 5) and the supplementary file (S1). The maximum Δ*K* indicates the best possible number of clusters that the genotypes can be grouped into.

## Discussion

The success of plant breeding programs depends mainly on the genetic variation of the plant breeding materials [24]. The farmer’s landraces are normally selected from the stocks available in different environments and they form a rich source of novel genes that can be used in breeding [25]. Analysis of variance of the phenotypic traits showed high variation in mung bean leaf traits. The high leaf petiole diameter observed in VC637245 genotype can be due to genetic variation causing increase in the cell size. The gene which controls petiole elongation, ROT3, if overexpressed, results in elongated petiole and leaf blades [26]. This serves as an adaptation of leaf exposure to light; hence, the variations recorded could therefore be due to adaptation to environmental conditions [27, 28]. Genotype VC614850 had the highest seed leaf length while genotype VC6137B had the lowest. Leaf features are therefore key in plant breeding since they are controlled by genes hence variation indicates genetic variations [29]. The current findings conform to those obtained in other diversity studies [30].

Genotype VC637245 had the lowest leaf diameter and this could be an adaptation to dry conditions since reduced leaf area reduces water loss via transpiration. This can also be attributed to loss of cell turgor, a common phenomenon in many plant species adapted to drought conditions [31]. The longer the length of leaves the higher the photosynthetic efficiency, and consequently, the higher the yield. This results due to increased photosynthetic assimilates, which manifest by high grain weight [32]. Genotype VC6173B was the tallest (32.98 cm), followed by KPS1 (31.920) while the shortest was VC6137B (22.66 cm). This variation recorded could be due to adaptation to wet and warm weather conditions, respectively. Plant height is crucial as it determines plants ability to take up carbon and, subsequently, its maturity. It is positively correlated to seed weight, grain yield, maturity, and hence its life span, a farmer preferred trait [33]. However, current findings differ with those of a similar study on mung beans, which recorded a range of 34-51 cm with a mean of 41.44 cm. This could be due to the variation in the geographical regions they were collected and the stage at which the measurements were taken [34]. A study by [31] indicated that the plants grown under drought conditions have a higher plant height range as compared to those growing under irrigated conditions. This could be due to drought conditions impairing cell division and expansion processes and eventually loss of cell turgor and hence the low growth rate, leaf area, plant height, and ultimately reduced plant yield [35].

Pod length contributed highly to genetic diversity among the mung beans studied (Table 2). This agrees with the findings by Kaur [36]. Plants adapted to humid areas produce a high number of pods and KPS1, N26, and VC61753B genotypes recorded a high number of pods. Pod length is strongly correlated to the grain weight, which subsequently affects plant yield [37]. This compares to the findings of a similar study on mung beans recorded a range of 27.5-15.0 pods per plant, which corresponds with the current findings [38]. However, they differed with the findings of Kumar [34]. Genotype VC61753B recorded the highest average seeds per pod per plant while VC6137B had the lowest. Reduced number of pods in a plant is an adaptation to dry conditions [38]. Research has shown a direct positive effect of the number of pods per plant on the seed yield [39]. This positive relationship between g weight and yield attributing traits has also been seen in pigeon peas [40]. Genotype VC637245 proved to be well adapted to stress conditions as it recorded the least seed weight among the studied genotypes. The low plant dry weight recorded in genotype VC637245 could be an adaptation to areas receiving low rainfall.

The genotypes that had high yield also recorded high harvest index N26, VC61753B, and VC6173B. Harvest index is determined by the mass of the specific tissue relative to the total biomass, and it decreases linearly with increasing drought in mung beans. Water is a key factor in mung beans biological yield and in wet environment the biological yield rises [41]. Biological yield has also been found positively and strongly related to plant height, days to flowering, and also pods per plant [37]. The improved varieties KPS1 and N26 took longer time to flower as compared to the land races but however recorded high number of nodes compared to the landraces. Plants growing in well-watered areas have delayed maturity and this subsequently affects the seed weight [42].

Cluster analysis of the seven mung bean genotypes showed a phenotypic distinction of the various species. The dendogram delineated the seven varieties into two distinct clusters with cluster I comprising four mung bean genotypes whereas cluster II comprised three genotypes. Grouping together of improved and local landraces in the two major clusters indicates they are likely to have a common origin. For example, N26, an improved genotype, and VC6173B showed a high similarity index of 0.7. The three genotypes in cluster II recorded high yield and harvest index thereof. The genetic distance clearly shows the genetic variations of the phenotypic traits of the mung bean plants in relation to the evolutionally history [30]. The three genotypes in cluster II were further clustered into two clusters, with VC61753B segregating itself. This confirms that the local landraces could be the parental genotypes to the improved variety. KPS1 in cluster I segregated itself to form its own sub-cluster which indicates its distant relationship and hence can be a suitable candidate in breeding programs.

Principal component analysis showed that the first three components contributed highly to the phenotypic variation and cumulatively accounted for 83.4% of the total variation among the studied traits. Lower principal component values were recorded in *V.radiata* study with the first three principal components explaining 70% of the total variation [43]. PCA analysis indicated the contribution of individual traits toward the phenotypic divergence of the mung bean genotypes. Among the studied traits, those that contributed highly to the variation include plant height, seed weight per plant, harvest index, biological yield, seeds per pod, seeds per plant, and pod length which are mainly the yield-contributing traits that are preferred by farmers. Genetic diversity studies on sorghum also gave similar findings, where the first and the second principal components accounted for 77.21% with the first principal component having 57.61%, which is slightly higher than the one observed in the current study. Leaf length, plant height, and plant yield contributed more to the phenotypic variation. Two genotypes, N26 and VC6173B, clustered closely in the scatter plot, indicating their genetic relatedness and conforming to the dendogram generated.

Polymorphic markers are ideal in plant breeding since they can discriminate between even closely related genotypes [44]. The basis for the use of molecular markers is mainly their polymorphism, which occurs naturally through natural mutation [45]. Microsatellite markers possess repeat regions of di-tri-tetra nucleotide sequences and sometimes more. The variation can be explained by replication slippage or unequal crossing over. SSR markers distinguish closely related lines hence were considered for this study, among other advantages like reproducibility.

The number of alleles (23) generated by the ten SSR markers analysis were shared among the studied varieties. The CEDG050 marker recorded the highest number of polymorphic alleles (5).

Rare alleles, highly informative alleles, were identified in this study. They can be used in DNA fingerprinting of mung beans and, consequently, test for genetic purity. A total of 23 alleles were detected in this study by the SSR markers and they ranged from 2 to 5 with an average of 2.875 alleles per locus. This gives the estimate of genetic diversity in the studied genotypes. This compares to the findings by Tangphatsornruang [20], who reported an allelic richness of 1-5 and an average of 3.01 alleles per microsatellite locus. However, findings of this study contrasted with those of Al-saady [46] which were relatively lower with an average of 2.14 per locus, respectively. In other diversity studies, higher values were obtained and this could be contributed by studying a diverse germplasm and also using a higher number of accessions [47]. Use of accessions from diverse origins can also contribute to such high allelic values.

The allele frequencies ranged from 0.4286 in CEDG050 marker to 0.7152 in CEDG056 marker with an average frequency of 0.5895. This was high as compared to the one obtained in mung beans studied by Molla [48]. This can be attributed to variations in the marker sequences, sample size, and the geographical origins of the genotypes studied. However, higher allele frequencies were recorded in China mung bean genotypes [49]. Null alleles were also recorded in some genotypes, where 20 loci had null alleles in 2-5 of the studied genotypes. This occurs when there is mutation at that specific locus hence primer binding cannot occur. Such findings have also been reported in other mung bean studies [50]. Rare alleles were also observed with the highest being recorded in CEDG050, which also recorded the highest number of alleles. The presence of rare alleles indicates a germplasm rich in genetic diversity [44]. The current findings indicate the accuracy of microsatelite markers in tracking the pedigree of mung beans breeding materials.

Polymorphic information content (PIC) of the markers ranged from 0.12 to 0.59 and this indicated the allelic diversity and the frequency among the mung bean genotypes. PIC values indicate if the markers are informative or not with a PIC value > 0.5 indicating high polymorphism and those with high PIC values can be contributed by very diverse varieties. PIC values ranging from 0.25-0.5 are considered moderately informative while those less than 0.25 are less informative [51]. Low PIC values could indicate that the varieties were closely related. The SSR markers showed a mean value of 0.3724, indicating their dependability in the study of diversity on mung beans [52]. Low PIC values can be due to gene frequencies concentration, thus deviating from the point of high polymorphism in a locus [44]. Markers with high frequency had low PIC values.

Gene diversity ranged from 0.5102 in CEDG010 to 0.1836in CEDG088 with an average of 0.3534. This is highly comparable to Gwag et al. (2010) and Somta et al. (2009) in their diversity studies on mung beans using microsatellite markers who obtained an average gene diversity of 0.345 and 0.39 respectively. However, it was slightly lower compared to 0.418 obtained by Lagat et al. (2016). The variation can be due to use of different primers and also the varieties used in each study. The moderate level of gene diversity within the studied *Vigna radiata* genotypes, which are self-pollinated, indicates their average genetic base. This can be due to the accumulation of combinations of novel genes as an adaptation to the natural selection pressures which keeps on varying [23].

Pairwise genetic dissimilarity analysis among the seven mung bean genotypes was also determined in this study using dissimilarity matrix with a range of 0.65. This indicates the rich diversity among the genotypes based on molecular analysis. In a similar study of mung beans using microsatellite markers, a similar range of 0.66 was obtained by Kaur et al. (2018). However, higher dissimilarity range of 0.85 was recorded by Kanavi et al. (2019). These variations in the levels of genetic dissimilarity could be due to selection of the same ancestors, similar traits or even intraspecific variations of the germplasm used. Genetic dissimilarity analysis of the seven mung bean varieties based on the 10 SSR markers indicated close relationship between genotypes N26 and VC637245. This could indicate common ancestry between the improved genotypes and local landraces. Variation was also recorded between the local landraces and the improved varieties. Genotype VC614850 was found to be distantly related to N26 and this indicates that they can be suitable candidates for breeding programs. The Fst values indicated moderate differences among the mung beans grown in the three regions.

The phylogenetic tree revealed genetic relatedness among the studied improved varieties and the local landraces. Cluster analysis of the seven mung beans based on the genetic similarity coefficients grouped them into three main clusters. This compares to the findings on other mung bean genotypes [12]. The improved varieties fell in the same clusters with the landraces, which indicates that they are genetically related. Genotypes VC6137B, N26, and VC637245 fell in the same cluster and this suggests that the three are genetically similar and have a common ancestry. Cluster II also comprised three varieties with KPS1 which is also an improved variety falling in this group. KPS1 usually have black pods and recorded a high number of pods per plant. The two improved varieties have been shown to be genetically diverse by genetic cluster analysis and grouping based on morphology. Similar observations were made on mung beans diversity studies by [53]. Bootstrap values were assigned to the clusters with cluster I having 65% while cluster II had 74%. In our study, 50% was considered statistically significant. The bootstrapping values at the cluster nodes act as indicators of variations among populations [54]. This agrees with findings on Indian mung beans [55].

Analysis of molecular variance (AMOVA) indicated that the main source of genetic variation was within populations, accounting for 99% of the total variation. This left an insignificant percentage of 1% to the variation among populations, which indicates that the *Vigna* genus has an insignificant variation among populations. This explains the low bootstrap values observed in the phylogenetic tree. Exchange of germplasm can also contribute to the very low genetic difference between populations. The high variation within populations could result from mutations which alter the repeats. The analysis of molecular variance findings differed with those done on mung beans using genome simple sequence repeat markers where 22% variation among populations was found and 78% resulted due to variance within populations [56]. Varieties selected from different countries recorded a high variation within populations of 51.6% and this could be because they are from diverse geographical locations [57].

The principal coordinate analysis (PCoA) indicates high variations among the varieties since the distances between the varieties show their variation. Varieties that are far from each other in the coordinates indicate high genetic distances and are hence distantly related. Those that are closely clustered like N26 and VC637245 are likely to be closely related and share the same traits. This conquers with the dendogram cluster analysis based on the genetic composition. This may also indicate that they are biological replicates. The values of principal coordinate 1 (37.52%) and principal coordinate 2 (28.53%) differed with those found on *Vigna ungiculata*, where the first three principal components explained 77.8% with the first component explaining the most (55.3%) [57].

Phenotypic variation was high based on the harvest indices but however clustering of the mung beans based on the phenotypic traits did not rhyme with the clustering based on their genetic composition. This can be due to the underrepresentation of the genome by the microsatellite markers or lack of association between the loci controlling the phenotypic traits and the molecular markers [58]. The disassociation can also be due to the agronomic traits being adaptive to the environment based on natural and artificial selection unlike the molecular markers. Marker trait association analysis revealed that seven of the thirteen agronomic traits studied associated with the microsattelite loci studied. These associations will be beneficial in future mung bean breeding programs.

## Conclusion

The current study indicates the genetic variation existing among mung bean genotypes in Eastern Kenya. Phenotypic traits such as seeds per pod, seed weight, and maturity profiles showed high variation among the genotypes and will help in breeding programs for yield improvement. Genetic advancement estimated for all the traits studied ranged from moderate to high. The polymorphism content among the eight SSR markers revealed that CEDG092 is the most informative and the others were reasonably informative. They can therefore be successfully applied to study genetic polymorphism and relationships among mung bean genotypes.

## Supplementary Information

**Additional file 1.** Supplementary file 1.

## Data Availability

The data used to support the current findings is included in this article. Any additional data will be availed by the authors upon request.

## Abbreviations

SSR: Simple sequence repeat
PC: Principal components
AMOVA: Analysis of molecular variance
RAPD: Random amplified length polymorphism
ISSR: Inter simple sequence repeats
STS: Sequence tagged repeats
AFLP: Amplified fragment length polymorphism
ANOVA: Analysis of variance
UPGMA: Unweighed pair group method with arithmetic mean
PCoA: Principal Component analysis
PCA: Principal component analysis
