# Supplementary material for: Genetic and phenotypic diversity of selected Kenyan mung bean (Vigna radiata L. Wilckzek) genotypes
Source: J Genet Eng Biotechnol. 2021 Sep 27;19:142. doi: 10.1186/s43141-021-00245-9 (PMC8476662; doi:10.1186/s43141-021-00245-9)
Supplement: Supplementary file 1 — Additional file 1. Supplementary file 1. [file 43141_2021_245_MOESM1_ESM.pdf]

	Reps	Mean LnP(K)	Stdev LnP(K)	Ln'(K)	|Ln''(K)|	Delta K
1	10	-221.340000	1.413585	—	—	—
2	10	-232.040000	17.734474	-10.700000	13.220000	0.745441
3	10	-229.520000	20.740181	2.520000	32.330000	1.558810
4	10	-259.330000	70.719965	-29.810000	59.120000	0.835973
5	10	-230.020000	20.906182	29.310000	25.710000	1.229780
6	10	-226.420000	7.879058	3.600000	4.160000	0.527982
7	10	-218.660000	2.853146	7.760000	13.660000	4.787697
8	10	-224.560000	11.462906	-5.900000	10.180000	0.888082
9	10	-220.280000	6.546551	4.280000	4.110000	0.627811
10	10	-220.110000	3.990670	0.170000	—	—

Supplementary file 1
